# Harnessing organelle engineering to facilitate biofuels and biochemicals production in yeast

**DOI:** 10.71150/jm.2501006

**Published:** 2025-03-28

**Authors:** Phuong Hoang Nguyen Tran, Taek Soon Lee

**Affiliations:** 1 Joint BioEnergy Institute, Emeryville 94608, CA, USA; 2 Biological Systems & Engineering Division, Lawrence Berkeley National Laboratory, Berkeley 94720, CA, USA

**Keywords:** yeast, organelle engineering, subcellular compartmentalization, metabolic engineering, biofuels and biochemicals

## Abstract

Microbial biosynthesis using yeast species offers numerous advantages to produce industrially relevant biofuels and biochemicals. Conventional metabolic engineering approaches in yeast focus on biosynthetic pathways in the cytoplasm, but these approaches are disturbed by various undesired factors including metabolic crosstalk, competing pathways and insufficient precursors. Given that eukaryotic cells contain subcellular organelles with distinct physicochemical properties, an emerging strategy to overcome cytosolic pathway engineering bottlenecks is through repurposing these organelles as specialized microbial cell factories for enhanced production of valuable chemicals. Here, we review recent progress and significant outcomes of harnessing organelle engineering for biofuels and biochemicals production in both conventional and non-conventional yeasts. We highlight key engineering strategies for the compartmentalization of biosynthetic pathways within specific organelles such as mitochondria, peroxisomes, and endoplasmic reticulum; involved in engineering of signal peptide, cofactor and energy enhancement, organelle biogenesis and dual subcellular engineering. Finally, we discuss the potential and challenges of organelle engineering for future studies and propose an automated pipeline to fully exploit this approach.

## Introduction

Microbial biofuels and biochemicals production offers several advantages over petroleum-derived production. For example, microbial bioproduction is not associated with the use of hazardous solvents, making it less environmentally detrimental. Likewise, efficient microbial conversion of cheap, abundant, and non-edible carbon streams including lignocellulosic biomass into fuels and chemicals has facilitated more sustainable bioproduction platforms (Okoro et al., 2022). With the capacity of scaling-up at industrial level (Crater & Lievense, 2018), biomanufacturing using microbial hosts provides a promising solution to meet the world’s energy demand while reducing dependence on fossil fuels.

Yeast species have been considered as a favorable eukaryotic host over prokaryotes such as *Escherichia coli*, for industrial applications owing to their comparative robustness, high tolerance to harsh environments, absence of phage contamination, and the availability of genetic engineering tools (Kim et al., 2020). Some species of yeasts are GRAS (“generally regarded as safe”) microorganisms and have become a chassis for biosynthesis of various products and for industrial applications (Kim et al., 2020). Presently, metabolic engineering efforts in yeast species have predominately relied upon cytosolic pathway optimization. For example, longstanding yeast metabolic engineering strategies for advanced biofuels and chemicals have focused on addressing the following challenges: limited cytosolic precursors (e.g. acetyl-CoA), competing pathways, spatial arrangement of multiple enzymes, metabolic cross-talks and toxicity of metabolites (Grewal et al., 2021; Lian et al., 2014; Qiu et al., 2018; Zhang et al., 2021). Given these challenges, organelle engineering has recently emerged not only as a potential approach to overcome limitations of cytosolic engineering but also as a unique engineering strategy characterized for eukaryotic systems.

**Fig. 1.**
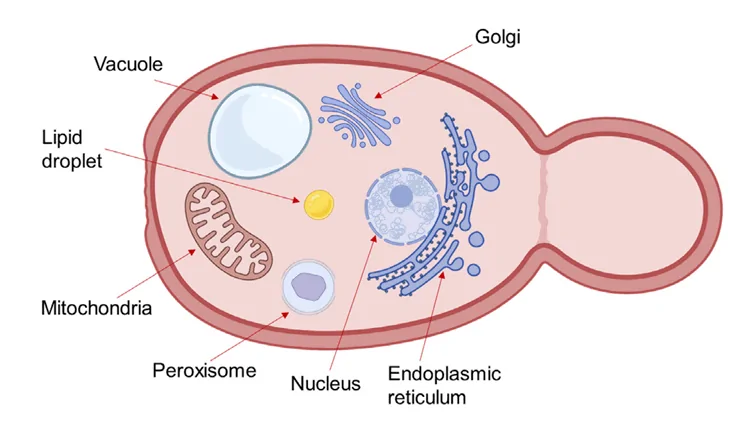
Primary cellular organelles found in yeasts (created in Biorender).

As eukaryotic organisms, yeasts are unicellular and have simple subcellular organization featuring organelles like the nucleus, endoplasmic reticulum (ER), Golgi apparatus, peroxisomes, mitochondria, and vacuoles (Fig. 1). These organelles are membrane-bound, with or without nucleic acids, and responsible for certain functions that support cell viability due to their inherent metabolism (Hammer & Avalos, 2017a). Organelle engineering involves compartmentalization of an entire or partial metabolic pathway to an organelle (Hammer & Avalos, 2017a). Successful organelle engineering depends on either aligning the native organelle metabolism with the introduced pathway or utilizing the subcellular membrane as a docking site to efficiently express specific enzymes and enhance the production of target products (Hammer and Avalos, 2017a; Yocum et al., 2022). Relative to cytosolic engineering, organelle engineering approaches showcased numerous improvements. Firstly, expressing a pathway within an organelle enhances local concentration of pathway substrates and enzymes to an environment where cofactors and precursors are generally more available (Hammer & Avalos, 2017a). Secondly, isolating a pathway to an organelle reduces interference by competitive/inhibitive pathways in the cytosol leading to the increased conversion rate of substrate to product (Huttanus & Feng, 2017). Conversely, if the products or byproducts of the introduced pathway are harmful to cells, isolation of the pathway to an organelle may lessen toxicity and improve cell growth (Hammer & Avalos, 2017a; Huttanus & Feng, 2017). Though organelle engineering offers a significant opportunity to improve yeast bioproduction, care should be taken while selecting the appropriate organelles for specialized pathways. Combinatory strategies should therefore be considered to balance global metabolism while maximizing the efficiency of the introduced biosynthetic pathway.

In this review, we summarize the advances of leveraging organelle engineering to improve biofuel and biochemical production in both conventional and non-conventional yeasts. In addition, we discuss the opportunities and challenges of metabolic engineering strategies on each targeted organelle. Subsequently, we assess the selection of specific organelles and suitable metabolic pathways from our perspective to identify the most effective approaches for organelle engineering.

## Mitochondrial Engineering

The yeast mitochondrion is an essential organelle regarded as the “powerhouse” of the cell. Mitochondria play an essential role in energy production via oxidative phosphorylation as well as for precursor and cofactor generation, which are crucial for cellular anabolism through the tricarboxylic acid (TCA) cycle. The mitochondrion has its own genetic machinery and multi-copy genomes and is encompassed by a selectively permeable double-layer phospholipid membrane (Malina et al., 2018). As mitochondria perform essential tasks associated with numerous cellular processes, mitochondrial engineering must carefully avoid any dysfunction that could negatively impact cell viability (Malina et al., 2018).

**Fig. 2.**
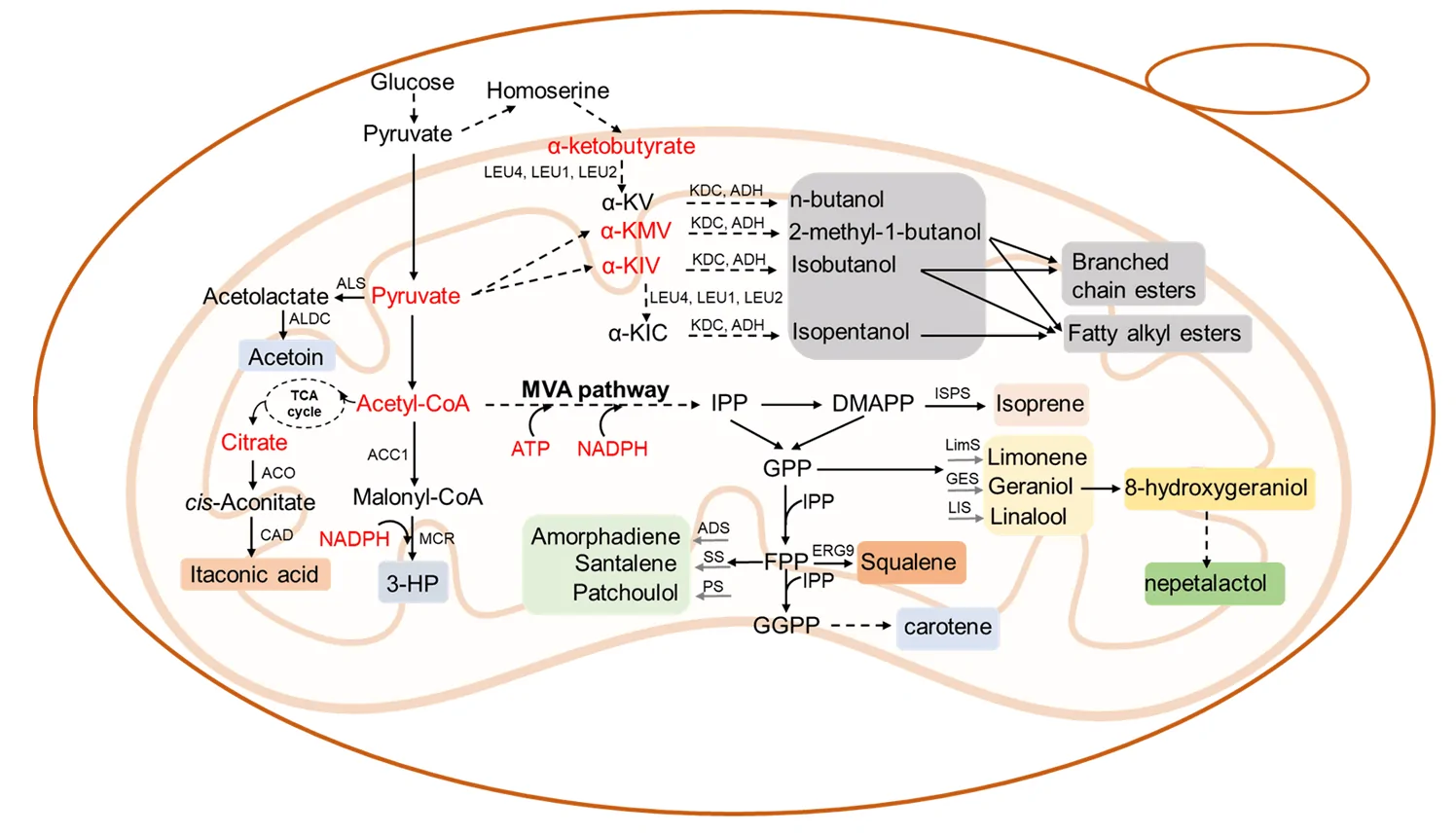
Repurposing mitochondria for biofuels and biochemicals production in yeasts. The available intermediates and cofactors in mitochondria are indicated in red. α-KV: α-ketovalerate, α-KMV: α-keto-β-methyl valerate, α-KIV: α-ketoisovalerate, α-KIC: α-ketoisocaproic acid, LEU4: α-isopropylmalate synthase, LEU1: isopropylmalate isomerase, LEU2: β-isopropylmalate dehydrogenase, KDC: α-Keto-acid decarboxylase, ADH: alcohol dehydrogenase, ALS: acetolactate synthase, ALDC: acetolactate decarboxylase, TCA: tricarbolic acid cycle, ACO: aconitase, CAD: cis-aconitate decarboxylase, ACC1: acetyl-CoA carboxylase, MCR: malonyl-CoA reductase, MVA: mevalonate, IPP: isopentenyl diphosphate, DMAPP: dimethylallyl diphosphate, GPP: geranyl diphosphate, FPP: farnesyl diphosphate, GGPP: geranyl geranyl diphosphate, ISPS: isoprene synthase, LimS: limonene synthase, GES: geraniol synthase, LIS: linalool synthase, ERG9: squalene synthase, ADS: amorpha-4,11-diene synthase, SS: santalene synthase, PS: patchoulol synthase.

Recent mitochondrial engineering in yeast has focused on isoprenoid production, especially of isoprene and terpenes (Hammer & Avalos, 2017a). As shown in Fig. 2, isoprenoids are produced in yeasts via the mevalonate (MVA) pathway. The MVA pathway starts with the condensation of two acetyl-CoA molecules to generate acetoacetyl-CoA with further conversion to ultimately generate the isoprenoid precursors isopentenyl diphosphate (IPP) and dimethylallyl diphosphate (DMAPP) (Lombard & Moreira, 2011). Engineering yeast mitochondria can facilitate isoprenoid biosynthesis because the concentration of acetyl-CoA, the fundamental precursor of the MVA pathway, is about 20 to 30-fold higher in mitochondria than that in cytosol where acetyl-CoA pool is both limited and drained by other competing pathways (Lian et al., 2014; Weinert et al., 2014). Likewise, the yeast mitochondrial matrix contains a surplus of reducing cofactors (NAPDH and NADH), and ATP molecules boosting reactions catalyzed by redox enzymes required in the MVA pathway (Hammer & Avalos, 2017a). As a result, previous engineering strategies for improved isoprenoid production in yeasts has traditionally centered on compartmentalizing the targeted isoprenoid pathway within mitochondria (Dong et al., 2021; Farhi et al., 2011; Yanagibashi et al., 2024a, 2024b; Yee et al., 2019; Yuan & Ching, 2016; Zhu et al., 2021). Up to 95% of mitochondrial proteins are synthesized in cytosol, then translocated to mitochondria due to an N-terminal mitochondrial targeting signal (MTS) (Dong et al., 2021), with or without cleavage of this MTS sequence to form mature proteins after import (Margeot et al., 2002). There are two widely-used MTSs which have been applied for mitochondrial engineering in yeast, COX4 encodes the subunit IV of the cytochrome C oxidase while COQ3 encodes an O-methyl transferase in coenzyme Q (Dong et al., 2021). However, there remains a knowledge gap about how many MTS sequences exist and what sequences facilitate the most efficient mitochondrial localization. In a prior study, Dong et al. (2021) performed a growth-based assay with confocal microscopy to screen twenty MTSs. The MTSs were derived from the most abundant mitochondrial proteins to direct non-native proteins, which included *E. coli* FabI and green fluorescent protein (eGFP), into mitochondria. In this work, they finally selected six out of twenty MTSs as the best candidates. Co-localizing the entire α-santalene biosynthetic pathway in *S. cerevisiae* mitochondria using these 6 well-performing MTSs resulted in 3.7-fold improvement in the α-santalene production (Dong et al., 2021). While several new MTSs were explored in the above study, further investigation of MTSs that efficiently direct heterologous proteins into the mitochondrion remains limited. To date, the bulk of localized mitochondrial expression has been conducted using the conventional MTSs from COX4 and COQ3.

Conventional MTS-tagged isoprenoid pathways in mitochondria have greatly improved production of various monoterpenes, triterpenes, sesquiterpenes in *S. cerevisiae* as listed in Table 1. Yuan & Ching (2016) reported a 63% improvement in amorpha-4,11-diene production compared to cytosolic production when the amorphadiene synthase (ADS) was mitochondria-targeted, suggesting that the mitochondrial matrix could be a better site for the ADS activity. Moreover, when the eight genes of farnesyl diphosphate (FPP) biosynthetic pathway were fully expressed in mitochondria, the amorphadiene titer reached 427 mg/L, 5-fold higher titer than that of replacing mitochondrial ADS by cytosolic ADS. This indicates that the mitochondrial membrane serves as a partial barrier to minimize the loss of the mitochondrial FPP to the cytosol where FPP can be consumed by competing pathways (Yuan & Ching, 2016). In addition, localization of the FPP pathway in mitochondria promoted the production of the sesquiterpenoid patchoulol by 2.71-fold compared to the production by the cytosolic pathway alone (Tao et al., 2022). Like FPP, yeast mitochondria were also reported to have rigid impermeability for geranyl diphosphate (GPP). Introduction of a GPP-derived geraniol biosynthetic pathway into yeast mitochondria led to 6-fold increase in geraniol production compared to cytosolic expression (Yee et al., 2019). The mitochondrial barrier for phosphorylated precursors of the isoprenoid pathway such as FPP and GPP has been considered as “a double-edged sword”. Though it could help to prevent loss of these precursors to competing pathways in the cytosol, the accumulation of phosphorylated intermediates inside yeast mitochondria can also cause impaired cell growth. Indeed, Yuan & Ching (2016) reported significant growth inhibition in the engineered strain expressing the mitochondrial FPP pathway. Inhibition was then mitigated upon overexpression of mitochondrial FPP consuming pathways. Metabolic burden and toxicity to yeast cells was also reported when isoprene and squalene synthesis pathway was compartmentalized in mitochondria (Lv et al., 2016; Yanagibashi et al., 2024a; Zhu et al., 2021). For example, Zhu et al. (2021) uncovered the detrimental effects of the accumulation of phosphorylated metabolites (e.g., mevalonate-5-phosphate (MVAP), mevalonate-5-diphosphate (MVAPP), and IPP/DMAPP) in the mitochondria with diphosphate molecules demonstrating more toxicity over their monophosphorylated counterparts. These diphosphorylated metabolites are converted into ATP analogues, which strongly inhibit several key components of the mitochondrial respiratory chain (Zhu et al., 2021). To tackle this challenge, a dual pathway engineering strategy was implemented. Here, a partial squalene pathway (from acetyl-CoA to DMAPP) was introduced into the mitochondria while the complete squalene pathway was expressed in the cytosol to simultaneously boost the squalene production (Zhu et al., 2021). In addition, they unexpectedly found that enhanced expression of truncated hydroxymethylglutaryl-CoA reductase (tHMG1) in cytoplasm could relieve the metabolic burden of mitochondrial engineering caused by accumulated toxic metabolites and effectively coordinate the dual pathway (Zhu et al., 2021). Yanagibashi et al. (2024a) also investigated the beneficial effects of fine-tuning dual pathways by controlling MVA pathway genes expression using promoters at various strengths. Additional examples of dual metabolic engineering strategy to enhance production of isoprenoids, including isoprene and limonene, are shown in Table 1 (Kong et al., 2023; Lv et al., 2016; Yao et al., 2018).

Mitochondrial engineering for 3-hydroxypropionate (3-HP) production has also been reported in *S. cerevisiae* (Zhang et al., 2023). Here, a mutant acetyl-CoA carboxylase (ACC1) and malonyl-CoA reductase (MCR) were engineered for mitochondrial localization using the COX4 signal (Fig. 2). Co-expression with a mitochondrial NADH kinase (POS5) and mitochondrial NADP-specific isocitrate dehydrogenase (IDP1) to increase mitochondrial NADPH availability enabled significant titer improvements. The dual-pathway engineered strain produced 3-HP titers of 6.16 g/L in shake flask and 71.09 g/L in a bioreactor, representing the highest 3-HP titer ever reported in yeast (Zhang et al., 2023).

As yeast mitochondria are primary sites for branched-chain amino acid (BCAA) biosynthesis, the production of advanced biofuels such as n-butanol, isobutanol, isopentanol, and 2-methyl-1-butanol has also been leveraged by directing enzymes involved in endogenous BCAA biosynthesis to the mitochondria (Fig. 2) (Avalos et al., 2013; Hammer et al., 2020; Shi et al. 2016). For example, mitochondrial compartmentalization of two enzymes in the Ehrlich pathway of valine/leucine/isoleucine (α-ketoacid decarboxylase (α-KDC) and alcohol dehydrogenase (ADH)) improved the isobutanol, isopentanol, and 2-methyl-1-butanol production from their precursor 2-ketoisovalerate (KIV) by 260%, 370%, and 500%, respectively (Avalos et al., 2013). To prevent early decarboxylation of KIV, Hammer et al. (2020) compartmentalized three enzymes related to leucine biosynthesis [2-isopropylmalate synthase (LEU4), isopropyl malate isomerase (LEU1) and β-isopropyl malate dehydrogenase (LEU2)] within mitochondria to elongate KIV to the second 2-ketoacid (KIC) using MTSs of COX4, CDC9, and COX6, respectively, while placing KDC and ADH in cytosol. This engineering approach led to a 31-fold increase in isopentanol production. Further deletion of competing genes that consume the intermediates pool resulted in 1.24 g/L of isopentanol titer (Hammer et al., 2020). A similar strategy was applied to increase threonine anabolism-based n-butanol production in *S. cerevisiae*. There, localization of LEU1, LEU4, LEU2, and LEU5, and a citramalate synthase (CimA) from *Leptospira interrogans* improved α-ketobutyrate pool in the mitochondria, yielding 1.05 g/L final titer with further optimization (Shi et al., 2016). Leveraging the high-level production of branched-chain alcohols by expressing ARO10 and ADH7 mitochondrially, Teo et al. (2015) showcased elevated production of fatty acid esters with short- and branched-chain alkyl groups. Yuan and Ching (2016) also reported efficient production branched-chain esters with additional expression of the native mitochondria targeted the alcohol acetyltransferase 1 (ATF1) (Teo et al., 2015).

Mitochondrial engineering in non-conventional yeasts has also been studied in *Aspergillus niger* and *Candida glabrata*. Blumhoff et al. (2013) for example, demonstrated a 14-fold and 24-fold increase in itaconic acid accumulation with mitochondrial expression of cis-aconitate decarboxylase (*Aspergillus terreus* CAD) and an aconitase (ACO) relative to cytoplasmic pathway in *A. niger*, respectively. These significant improvements were characterized by robust production of citric acid, a precursor of the itaconic acid pathway (Fig. 2) (Blumhoff et al., 2013). In *C. glabrata*, the acetoin pathway comprising acetolactate synthase (*Bacillus subtilis* ALS) and acetolactate decarboxylase (*Bacillus amyloliquefaciens* ALDC) were localized in the mitochondria (Fig. 2). When coupled with upregulation of mitochondrial pyruvate carrier (MPC), the engineered strain generated 59.8% more acetoin compared to the cytoplasmic pathway (3.26 g/L vs. 2.04 g/L) (Li et al., 2015).

As mitochondria can serve as a metabolic center for the production of biofuels and biochemicals in yeast, enlargement of mitochondria is another promising strategy in mitochondrial engineering. However, only one study to date has investigated mitochondrial morphology manipulation. Yanagibashi et al. (2024b) discovered deletion of *MDM32*, a mitochondrial morphology-related gene, could increase mitochondrial volume in *S. cerevisiae*. Deletion of MDM32 improved squalene and β-carotene titers by 2.8-and 1.4-fold, respectively.

## Peroxisomal Engineering

**Fig. 3.**
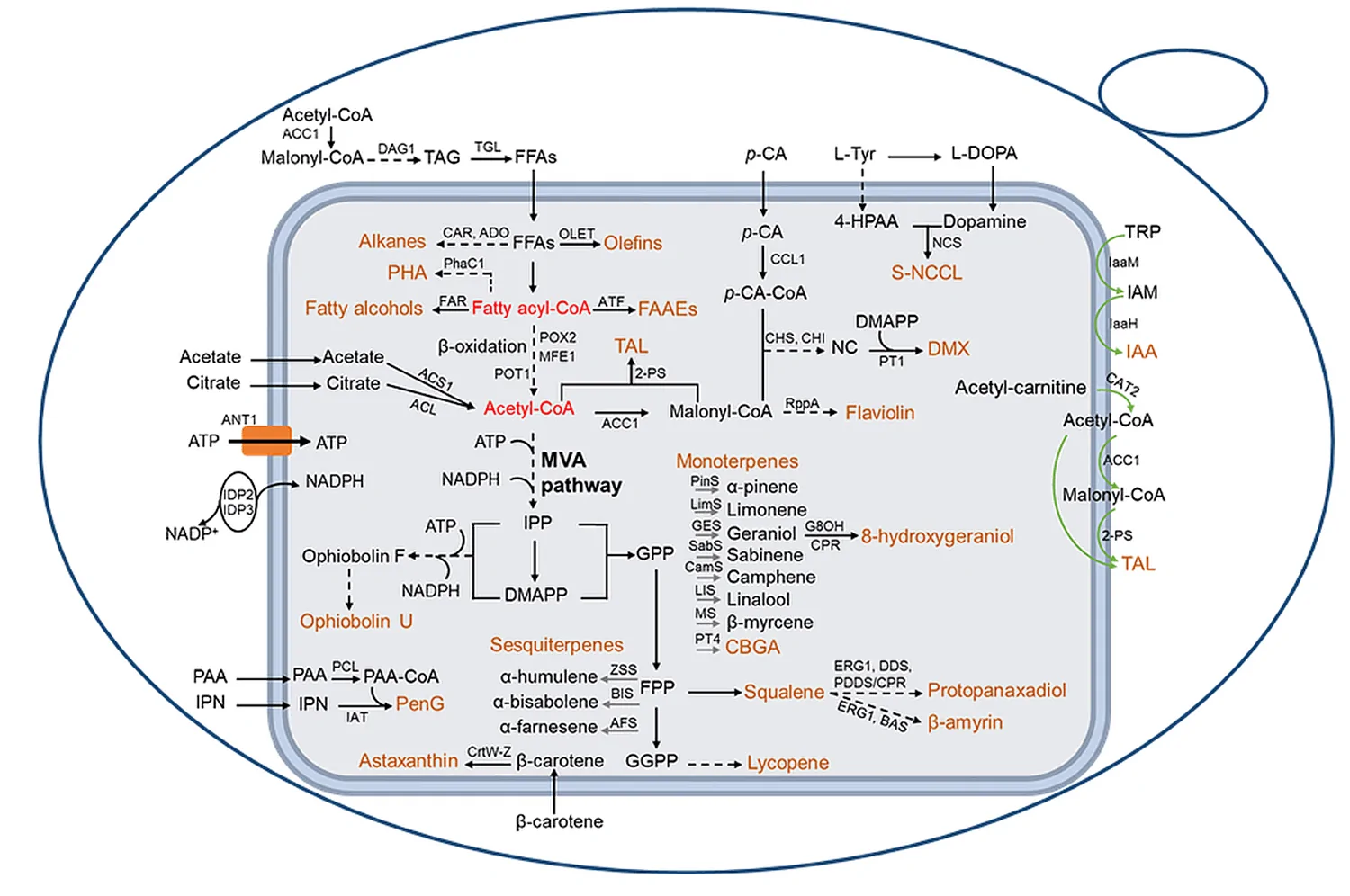
Harnessing peroxisomes for biofuels and biochemicals production in yeasts. Metabolites in red indicate key intermediates for biosynthesis in the peroxisome. Green arrows indicate bioconversion by peroxisomal surface docking enzymes. FFA: free fatty acids, DAG1: diacylglycerol acyltransferase, TGL: triglyceride lipase, CAR: carboxylic acid reductase, ADO: aldehyde deformylating oxygenase, OLET: Terminal Olefin, P450 fatty acid decarboxylase, PhaC1: PHA synthase, FAR: fatty acyl reductase, ATF: alcohol acetyl transferase, POX2: acyl-CoA oxidase 2, MFE1: multifunctional enzyme type-1, POT1: 3-ketoacyl-CoA thiolase, ACS1: acetyl-CoA synthetase 1, ACL: ATP citrate lyase, ANT1: adenine nucleotide translocase 1, IDP2-IDP3: NADP-dependent isocitrate dehydrogenase isoenzyme, 2-PS: 2-pyrone synthase, RPPA: 1,3,6,8-tetrahydroxynaphthalene synthases, *p*-CA: *para*-coumaric acid, CCL1: 4-coumarate-coenzyme A ligase, CHS: chalcone synthase, CHI: chalcone isomerase, PT1: prenyltransferase 1, 4-HPAA: 4-hydroxyphenylacetaldehyde, NCS: norcoclaurine synthase, S-NCCL: S-norcoclaurine, TRP: tryptophan, IaaM: tryptophan-2-monooxygenase, IAM:indole-3-acetamide, IaaH: IAM hydrolase, IAA: indole-3-acetic acid, TAL: triacetic acid lactone, PinS: pinene synthase, SabS: sabinene synthase, CamS: camphene synthase, MS: myrcene synthase, PT4: geranyl transferase 4, G8OH: 8-hydroxygeraniol synthase, CPR: cytochrome P450 reductase, ZSS: α-humulene synthase, BIS: bisabolene synthase, AFS: α-farnesene synthase, ERG1: squalene epoxidase, CrtW-Z: fusion enzyme of β-carotene ketolase and hydroxylase, DDS: dammarenediol II synthase, PDDS: protopanaxadiol synthase, BAS: β-amyrin synthase, PCL: phenylacetyl CoA ligase, IAT: isopenicillin N-acyl transferase.

The peroxisome is a ubiquitous DNA-free and single bilayered organelle in yeasts. It is involved in numerous metabolic processes and functions, including hydroperoxide formation, β-oxidation of fatty acids, the glyoxylate shunt, and methanol metabolism (Sibirny, 2016). There is a growing focus on compartmentalizing heterologous acetyl-CoA utilizing pathways into the peroxisome because β-oxidation provides an abundant acetyl-CoA pool. Unlike mitochondria, peroxisomes are not essential for cell growth. As a result, peroxisome engineering is less impactful to cellular fitness and holds more potential to serve as a microfactory for biofuels and biochemicals production. In addition, metabolites can passively diffuse or be transported through the single membrane of the peroxisome. The peroxisome was reported to be a suitable subcellular location providing a better physicochemical environment than other sublocations for expression of rate-limiting enzymes involved in terpenoid biosynthesis (Shu et al., 2024). Therefore, peroxisome engineering has been widely implemented as an effective approach for synthesizing many value-added chemicals across various species of yeasts including monoterpenes, sesquiterpenes, triterpenes, carotenoids, polyketides, alkaloids, alkane, alkene, polyhydroxyalkanoates (PHA), fatty alcohols, amino acids and antibiotics (Table 2, Fig. 3). In this section, we showcase engineering strategies for maximizing the capacity of the peroxisome as a depot for heterologous biosynthetic pathways.

### i) Enhancing importing rate of proteins into peroxisomes by engineering peroxisomal targeting sequences (PTS)

Peroxisomal targeting sequences (PTS) for peroxisomal soluble proteins in yeasts are classified into two groups, PTS1 and PTS2 (Sibirny, 2016). In PTS1, a signal peptide at the C-terminus is recognized by the PEX5 cargo receptor protein. All evolutionarily diverse sequences of PTS1 share a consensus motif as (S/A/C)-(K/R/H)-(L/M). In *S. cerevisiae*, PTS1’s are mostly known as SKL (Serine-Lysine-Leucine). A majority of peroxisomal matrix proteins possess PTS1 whereas a minority use PTS2. In PTS2, an N-terminal signal peptide with a conserved sequence of (R/K)-(L/V/I)-X_5_-(H/Q)-(L/A) uses PEX7 as a cargo receptor. Finally, most peroxisomal membrane proteins are targeted by a membrane PTS (mPTS) and recognized by the PEX19 receptor (Sibirny, 2016).

PTS is crucial to compartmentalization of a target pathway into the peroxisome. Modifying pathway genes with the canonical PTS1 C-terminus SKL signal peptide improved monoterpene, sesquiterpene, and triterpene production by several to over 100-fold among yeast species (Table 2), suggesting that peroxisomal engineering offers an easily applicable approach for enhancing biochemical production. Residues upstream of the SKL signal peptide were suggested to impact the recognition of the PEX5 receptor (DeLoache et al., 2016). As a result, a library of randomized PTS1 peptide linker sequences was created to optimize peroxisomal protein import. By screening the library using a novel enzyme-based assay, DeLoache et al. (2016) discovered that positively charged residues significantly enhanced PTS1 import into the peroxisome in *S. cerevisiae*. This newly identified signal peptide “ePTS1” (LGRGRR-SKL) was then used in numerous studies for efficient compartmentalization of various biosynthetic pathways in peroxisomes, leading to titer improvement not only in *S. cerevisiae* but also in *Pichia pastoris* (Table 2). In contrast to ePTS1, Lin et al. (2023) discovered that “pPTS1” (TFAKSSRNK-SKL), a PTS1 derivative selected from oxalyl-CoA synthetase PCS60 for peroxisomal targeting of *Gerbera hybrida* type III 2-pyrone synthase (Gh 2-PS), improved TAL titer by 3.64-fold. The improvement suggests that pPTS1 might serve as an efficient signal peptide for polyketide production (Lin et al., 2023). Furthermore, Zhang et al. (2024) an orthogonal peroxisomal transport system ScPEX5*-oPTS1* that includes an artificial transporter PEX5* (a fused protein of N-terminal ScPex5 and C-terminal *Arabidopsis thaliana* Pex5p domains) and optimized signaling peptide “oPTS1*” (YERVTTMTNYQ-SYY) obtained through pigment-based high-throughput screening (HTS) in *S. cerevisiae*. This novel system demonstrated better transportation efficiency, resulting in 39% and 80% increase of mevalonate production compared to the PEX5-ePTS1 and PEX5-PTS1 systems, respectively, while ultimately improving α-humulene production to 17.33 g/L (Zhang et al., 2024).

Non-conventional yeasts grow on altered carbon substrates, which may influence the expression of numerous genes involved in peroxisome metabolism such as peroxisomal receptor proteins. Therefore, native and engineered PTSs that have been developed and characterized in *S. cerevisiae* may be limited in application for the peroxisomal targeting of biosynthetic pathways in non-model yeasts. Ye et al. (2024) harnessed a HTS approach to screen 25 putative PTS1’s in *P. pastoris*, then performed directed evolution of the selected PTS1’s to improve the targeting efficiency. Additionally, tagging a bisabolene synthase (BIS) with the LARF peptide found in alcohol oxidase (AOX) resulted in a higher production of α-bisabolene than that tagged with SKL in *P. pastoris* (Gao et al., 2024b).

Despite relatively limited use, PTS2 has been applied as an efficient signal peptide for peroxisomal compartmentalization of fatty alcohol and olefin biosynthetic pathways in *S. cerevisiae* (Zhou et al., 2016) as well as the limonene pathway in *Rhodotorula toruloides* (Gao et al., 2024a). Furthermore, the combination of different PTS’s has been investigated for subcellular localization of biosynthetic pathways to enhance bioproduction. For example, ePTS1 and PTS1_TAL1-2 _were used for localizing the geraniol pathway in *P. pastoris* whereas SKL and LARF were used for isolating the α-bisabolene pathway to peroxisomes (Gao et al., 2024b; Ye et al., 2024). Moreover, PTS1 and PTS2 were co-employed as peroxisomal targeting signals for compartmentalizing the limonene biosynthetic pathway in *R. toruloides* (Gao et al., 2024a) protopanaxadiol and alkane biosynthetic pathway in *S. cerevisiae* (Choi et al., 2022; Zhou et al., 2016) α-olefines and amino acids biosynthetic pathway in *P. pastoris* (Cai et al., 2022) and *Schizosaccharomyces japonicus* (Gu et al., 2023), respectively.

### ii) Increasing acetyl-CoA, energy and cofactors required for the biosynthetic pathways in peroxisome

Peroxisomes can serve as good metabolic hubs of various acetyl-CoA-derived bioproducts; however, challenges remain in utilizing them as powerful subcellular factories. For example, peroxisomal acetyl-CoA availability is much lower compared to the mitochondria as non-oleaginous yeasts like *S. cerevisiae* typically do not exhibit a robust fatty acid β-oxidation pathway. In addition, energy and cofactors, which are required for heterologous biosynthetic pathways like the MVA pathway, are low relative to the mitochondria. Hence, increasing the acetyl-CoA, ATP, and NADPH pools would help to reach high-level production of bioproducts in peroxisome.

To this end, the overexpression of peroxisomal adenine nucleotide transporter (ANT1) has been used to elevate the peroxisomal ATP levels via the transport of cytosolic ATP to the peroxisome (Fig. 3) and has significantly improved α-humulene production in *Y. lipolytica* (Guo et al., 2021, 2022; Liu et al., 2020; Zhang et al., 2024). Providing adequate level of essential cofactors such as NADPH to the peroxisome could also be addressed by upregulating the isocitrate-2-oxo-glutarate redox shuttle (cytosolic and peroxisomal isocitrate dehydrogenases (IDP2 and IDP3, respectively)) which is responsible for circulating NADP/NADPH between the cytosol and peroxisome (Fig. 3). Implementing a combined strategy by overexpressing ANT1, IDP2, and IDP3 led to an enhanced production of α-humulene and squalene in *S. cerevisiae* (Liu et al., 2020; Zhang et al., 2024).

The majority of acetyl-CoA in the peroxisome is derived from β-oxidation. Upregulation of β-oxidation by overexpressing genes involved in fatty acid degradation pathway such as POT1, POX2, and MFE1 has been reported to facilitate sesquiterpene and triterpene production in yeasts (Fig. 3) (Guo et al., 2021, 2022; Ma et al., 2024). In addition, Ma et al. (2024) enhanced TAG-derived free fatty acids level by overexpressing a lipase encoded by *tgl* from *Thermomyces lanuginosus* along with a diacylglycerol acyltransferase (DAG1) and an acetyl-CoA carboxylase (ACC1) (Fig. 3) to make β-oxidation more dominant in yeast, increase acetyl-CoA accumulation, and ultimately improve squalene production titer.

As the peroxisomal membrane is impermeable to acetyl-CoA, cytosolic acetyl-CoA is unable to be used directly in peroxisomal biosynthesis. However, intermediates such as acetate and citrate can diffuse into peroxisome. Along this thread, strategies to improve acetyl-CoA level from acetate and citrate in the cytosol have been pursued (Fig. 3). For example, Liu et al. (2020) localized acetyl-CoA synthetase (ACS1) and ATP-dependent citrate lyases (ACL1 and ACL2) of *Y. lipolytica* in the peroxisome and observed that the peroxisomal acetyl-CoA level increased by converting acetate and citrate that entered the peroxisome from cytosol. This engineering contributed to improved squalene bioproduction (Liu et al., 2020). In addition, Ma et al. (2024) introduced a feedback-insensitive acetyl-CoA synthetase from *Salmonella enterica* (SeACS1^L641P^) into the peroxisome and improved the squalene titer by 63%.

### iii) Improving peroxisome biogenesis and enlarging peroxisome functional capacity

**Fig. 4.**
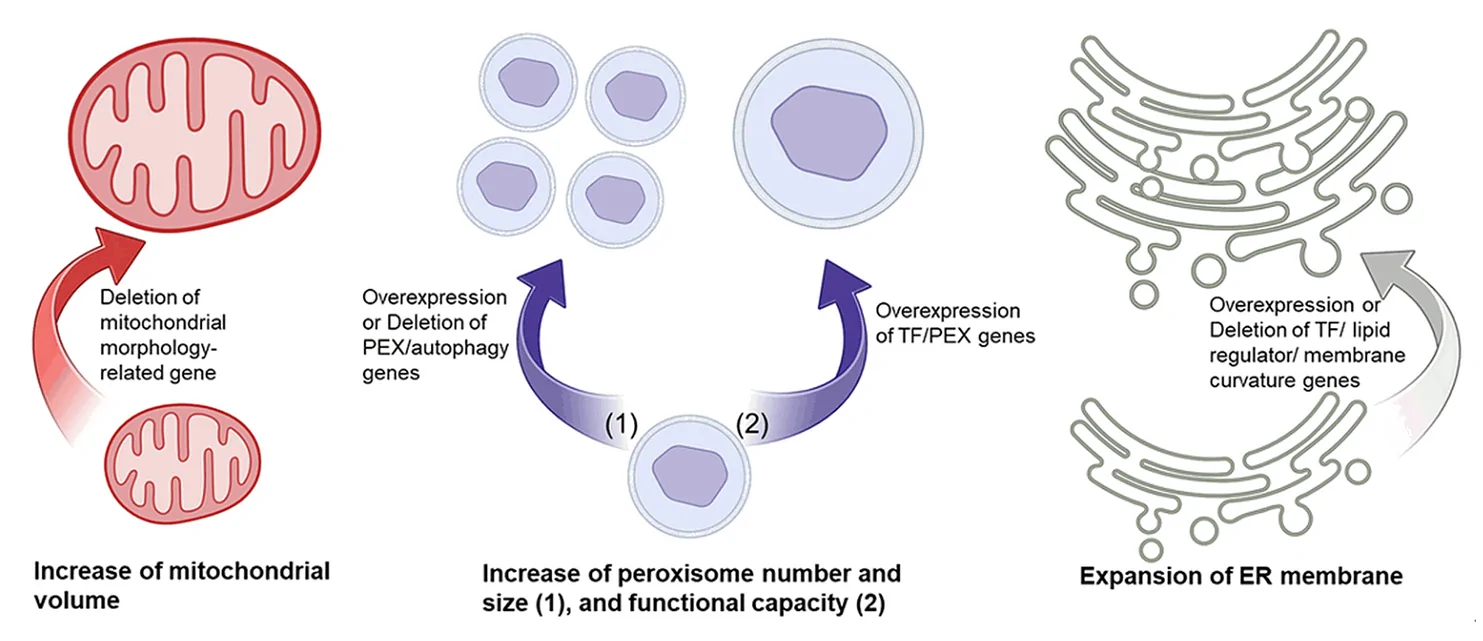
Improving compartmentalization capability of subcellular organelles through morphology and functional capacity-related engineering (created in Biorender). TF: transcriptional factor, PEX: peroxin.

Engineering strategies for facilitating peroxisome biogenesis predominantly pertain to improving (1) the rate of targeted protein import into the peroxisome and (2) peroxisome proliferation (number and size) (Fig. 4). Proteins encoded by PEX genes that play a role in the regulation of peroxisome biogenesis are called “peroxins”. Overexpression of protein receptors (PEX5 or PEX7) corresponding to the signal peptide (PTS1 or PTS2) could improve the import of targeted matrix proteins. Sheng et al. improved fatty alcohol production in *S. cerevisiae* by 37.1% when PEX7 was overexpressed along with fatty acyl-CoA reductase tagged with PTS2 (Sheng et al., 2016). Similarly to PEX7, coordinating expression of PEX5 could increase mevalonate production by ePTS1-tagged pathway in *S. cerevisiae* (Zhang et al., 2024) and also affect amino acid biosynthesis in a non-model *S. japonicus*. In the case of engineering biosynthetic pathway docking on peroxisomal membrane surface (discussed in the next section), Bassett et al. (2024) demonstrated the improved production of indole-3-acetic acid (IAA) of *Kluyveromyces marxianus* engineered with an anchoring pathway using C-terminal docking domain from *S. cerevisiae* PEX15. Overexpression of native PEX19 responsible for membrane-docking and translocation in *K. marxianus* potentially contributed to larger membrane docking sites and resulted in approximately 1.75-fold increase in IAA production (Bassett et al., 2024).

Peroxisome proliferation is defined by the intracellular peroxisome population and size. Regulating peroxisome proliferation through peroxins could be an effective approach for improved peroxisomal production of targeted biochemicals. As mentioned above, overexpression of dual-function proteins PEX3 and PEX19 improved fatty alcohol production (Sheng et al., 2016). Additionally, Zhou et al. (2016) enhanced peroxisome biogenesis through deletion of peroxins PEX31 and PEX32, leading to more and larger peroxisomes. Proliferation resulted in 77% and 25% higher peroxisomal production of fatty alcohols and alkanes, respectively, in *S. cerevisiae*. Furthermore, when PEX34 was overexpressed and coupled with a PEX31 and PEX32 knock-out, alkane production was remarkably increased by 54%, representing 7-fold higher titer than that of cytosolic pathway (Zhou et al., 2016). Likewise, deletion and/or overexpression of peroxins controlling peroxisome division was applied to improve production of fatty alcohols (PEX3, PEX19 overexpression) (Sheng et al., 2016), geraniol (PEX30 and PEX32 deletion) (Gerke et al., 2020), protopanaxadiol (PEX11 deletion, PEX34 overexpression) (Choi et al., 2022) in *S. cerevisiae*; squalene (PEX10 overexpression) in *Y. lipolytica* (Ma et al., 2024); and triacetic acid lactone (TAL) (PEX11 overexpression) in *K. marxianus* (Bassett et al., 2024). There are also non-peroxin proteins which were revealed to have an impact on peroxisome proliferation. For example, pexophagy related proteins trigger the selective degradation of peroxisomes via autophagy. Hence, deletion of these proteins such as ATG36 and ATG17 could stabilize the number and reduce the turnover of peroxisomes by preventing degradation, as shown by improved production of protopanaxadiol in *S. cerevisiae* (Choi et al., 2022) and TAL in *K. marxianus* (Bassett et al., 2024). Moreover, Bassett et al. (2024) reported that upregulation of DNM1 and PHO85 was beneficial on specific production of IAA and TAL in *K. marxianus*. DNM1 is a dynamin-associated protein involved in peroxisome fission after elongation while PHO85 is a kinase that acts as a regulator of PEX11 (Bassett et al., 2024).

When *S. cerevisiae* is grown in glucose-containing medium, the peroxisome appears small with the average diameter of 0.2 μm, though can be enlarged to 0.3–0.5 μm in oleate-supplemented medium (Vizeacoumar et al., 2004; Yofe et al., 2017). Yet, growth of yeast cells in the presence of oleate was found to be poor (Grillitsch et al., 2011) and causes impaired production. As a result, Grewal et al. (2021) focused on engineering three transcriptional factors (ADR1, OAF1, and PIP2), which control peroxisome proliferation but are repressed by glucose, to enlarge functional capacity of the peroxisome and thereby enhance biochemical production on glucose without oleate induction (Fig. 4). Overexpression of a mutated ADR1^S230A^ and regulatory domain removal of OAF1 and PIP resulted in peroxisome proliferation, creating larger peroxisomes and reducing degradation of cargo proteins (Grewal et al., 2021), which led to improvements of (*S*)-norcoclaurine and TAL production by 47% and 46.5%, respectively (Grewal et al., 2021; Lin et al., 2023). Advancements of computational algorithms and artificial intelligence (AI) have made machine learning (ML) a powerful tool for conducting extensive screening of peroxisome complexes. These screenings aim to identify candidates that genuinely enhance the functional capacity of peroxisome in *S. cerevisiae* without disrupting their native functions (Baker et al., 2024). By implementing an ML-guided approach, Baker et al. (2024) explored a massive combinatory space that included expression of all peroxisome related genes. They found that the EPC (enhanced peroxisome capacity) strain, which overexpressed peroxisome-related protein complexes such as PEX2, PEX5, PEX6, PEX8, PEX10, PEX15, PEX17, and PEX22, had the highest peroxisome capacity and outperformed strains with rational overexpression of peroxisome complexes or transcriptional factors (TFs) by 39% or 21%, respectively. This EPC strain demonstrated an 80% improvement in geraniol production, producing 0.957 g/L in batch and 9.5 g/L of geraniol in fed-batch fermentation (Baker et al., 2024).

### iv) Protein docking on peroxisomal surface

Peroxisomal surface display has recently emerged as a successful approach for harnessing untapped peroxisomal acetyl-CoA to improve biosynthesis. Unexploited acetyl-CoA derived from the β-oxidation pathway in the peroxisome is exported for successive utilization in the mitochondria in the form of acetyl-carnitine catalyzed by mitochondrial/peroxisomal carnitine acetyl-CoA transferase encoded by CAT2 (van Roermund et al., 1999). Encouraged by this conversion, Yocum et al. (2022) used a C-terminus docking signal peptide from *S. cerevisiae* PEX15 to express CAT2, ACC1^S1157A^, and Gh2-PS on the surface of the peroxisomes (Fig. 3). They saw a 15.5-fold improvement in TAL production from the multi-copy anchored TAL pathway compared to a low-copy cytosolic 2-PS expression in *S. cerevisiae*. Likewise, in *K. marxianus*, Bassette et al. (2024) saw a 100% increase of TAL titer when using this triple enzyme cascade along with a 7.9-fold titer improvement of IAA with peroxisomal surface display of *Pseudomonas savastanoi* tryptophan-2-monooxygenase (IaaM) and indole-acetamide hydrolase (IaaH) using *S. cerevisiae* PEX15 docking peptide (Fig. 3). Recently, Zhang et al. (2024) improved cytosolic acetyl-CoA pool for ophiobolin production by docking CAT2 to the peroxisomal membrane to directly obtain free acetyl-CoA from peroxisomal acetyl-carnitine export.

### v) Harnessing dual cytoplasmic-peroxisomal pathway engineering for improved bioproduction

Unless the metabolites generated from the biosynthetic pathway are toxic to the cells, harnessing dual regulation of cytoplasm and peroxisome would significantly enhance production of biochemicals. This approach, either through mating haploid peroxisomal/cytoplasmic strain to form a diploid strain or developing both peroxisomal and cytoplasmic pathways in a single haploid strain, has been widely applied for the biosynthesis of terpenoids as shown in Table 2. The native cytosolic MVA pathway, a crucial component of the terpenoid pathway, plays an essential role in yeasts by supplying the FPP precursor for the biosynthesis of ergosterol, an important molecule for membrane integrity. Thus, integrating a peroxisomal pathway in addition to the native MVA pathway could maximize potential terpene production. For instance, dual pathway expression in the cytoplasm and peroxisome yielded higher production of limonene at 1.05 g/L in *R. toruloides* (Gao et al., 2024a); linalool and β-amyrin at 2.6 g/L in *S. cerevisiae* (Du et al., 2022; Zhou et al., 2023); α-humulene at 17.33 g/L and 21.7 g/L in *S. cerevisiae* (Zhang et al., 2024) and *Y. lipolytica* (Guo et al., 2022), respectively; α-farnesene at 2.56 g/L in *P. pastoris* (Liu et al., 2021); and squalene at 11 g/L and 32.8 g/L in *S. cerevisiae* (Liu et al., 2020) and *Y. lipolytica* (Ma et al., 2024), respectively.

## Endoplasmic Reticulum (ER) Engineering

The endoplasmic reticulum (ER) is a peripheral organelle extending from the nuclear envelope and harboring a membrane network of interconnected tubules and cisternae in a single lumen (West et al., 2011). As the ER is close to the plasma membrane in yeast, it can be referred to as the cortical ER. There are three major domains of yeast ER; the plasma membrane-associated ER, the central cisternae ER, and the tubular ER (West et al., 2011). The ER functions primarily as a microenvironment for protein synthesis, folding, and exporting involved in the secretory pathway and is the predominant site of membrane lipid biosynthesis (e.g., phospholipids) and calcium storage (Schwarz & Blower, 2016). Unlike mitochondria and peroxisomes, which are well-studied for synthetic biology applications, research on ER engineering has only recently been explored and mainly focuses on either (i) harnessing the ER as a subcellular location for an ER- non-native pathway shortcut or (ii) enlarging ER capacity for efficient expression of membrane/ER-associated proteins and storage of lipid-related bioproducts (Fig. 4).

Morphine, an opiate molecule, belongs to a family of benzylisoquinoline alkaloids (BIAs) that are widely used as painkilling medications. Production of morphine from thebaine in *S. cerevisiae* utilized three heterologous enzymes from *Papaver somniferum*; including 2-oxoglutarate/Fe^2+^-dependent dioxygenases T6ODM and CODM, and NADPH-dependent aldo-keto reductase COR (Thodey et al., 2014). Specifically, T6ODM converted thebaine to neopinone which was then spontaneously transformed to codeinone, followed by reactions catalyzed by COR and CODM to generate codeine and morphine, respectively. Upon introduction of the morphine biosynthetic pathway into yeast, Thodey et al. (2014) discovered an undesired pathway generating the nontarget neomorphine due to promiscuous activity by COR on the substrate neopinone. By localizing COR in the ER using a C-terminal routing peptide tag from CNE1, conversion of cytoplasmic neopinone to neomorphine by COR was limited and production of morphine increased to 3.1 mg/L with higher specificity (86%) compared to the cytosolic COR expressing strain (2.5 mg/L and 44% specificity) (Thodey et al., 2014). These results suggested that the ER could be harnessed as an insulating lumen to prevent the access of specific enzymes to unwanted substrates and thus elevate the reaction specificity towards the target product.

Moreover, the ER has been previously shown to be an optimal subcellular location for heterologous expression of membrane-associated enzymes such as human sphingolipid desaturase (hDES1) in yeast (Murakami et al., 2015). hDES1 was reported to have multiple transmembrane domains and supposed to be an integral membrane protein. Encouraged by this insight and the observation that several sphingolipid desaturases in monkeys and yeast are localized in the ER, Murakami et al. (2015) demonstrated that targeting hDES1 to the ER using the retention signal KKEK resulted in 2-fold increase of ceramide-NS production relative to cytosolic hDES1. Their work realized the potential of the ER for efficient expression of non-native transmembrane proteins in yeast.

In addition, the ER can be applied to the biosynthesis of lipophilic compounds such as carotenoids and fatty acid-derived chemicals. Ma et al. (2021) reported an enhanced production of astaxanthin production through targeting the fusion enzyme (CrtW-Z) of *Paracoccus* sp. β-carotene ketolase (PsCtrW) and *Haematococcus pluvialis* hydroxylase (HpCrtZ). The strain harboring ER-targeted CrtW-Z yielded 53.2 mg/L of astaxanthin, demonstrating a 1.84-fold increase relative to the cytosolic pathway (Ma et al., 2021). Xu et al. (2016) employed the ER as a metabolic depot for production of fatty acid ethyl esters (FAEEs), fatty alkanes, and fatty alcohols in *S. cerevisiae*. Using the peptide KDEL as the ER signal and targeting wax ester synthase sourced from *Acinetobacter baylyi* ADP1 (AbATFA), FAEE production reached 136.5 mg/L, which is a 15-fold improvement compared to cytoplasmic expression of AbATFA. Surprisingly, the ER-AbATFA engineered strain yielded longer chain FAEEs compared to when AbATFA was targeted to the peroxisome (Xu et al., 2016). Similarly, overexpression of the ER-targeted gene cluster consisting of fatty acyl-CoA reductase (AbACR1) and *Prochlorococcus marinus* aldehyde deformylating oxygenase (PmADO) improved alkane production to 16.8 mg/L (Xu et al., 2016). Furthermore, compartmentalizing two enzymes of fatty alcohol synthesizing pathway, fatty acyl-CoA reductase (ACR1) and aldehyde reductase (AHR) from *A. baylyi* and *E. coli*, respectively, facilitated the production of fatty alcohols with a titer of 49.2 mg/L (Xu et al., 2016). Given the increased production of FAEEs and fatty alkanes with ER-targeted enzymes, it is possible that the ER could serve as a microfactory for fatty acid-derived bioproducts. Since biochemicals such as FAEEs are transported within the cell mainly through lipid droplets (LDs) which are branched from the ER, compartmentalizing the downstream FAEE biosynthesis pathway in the ER might improve the partitioning of synthesized FAEEs to LDs, thereby enhancing production (Xu et al., 2016).

A study in yeast by Jiao et al. (2024) demonstrated that the ER not only provides a favorable microenvironment for enzyme activity regarding pH, but the C-terminal ER signal peptide could increase enzyme stability as well. (*S*)-Scoulerine ((*S*)-SCO), a key intermediate of the protoberberines and benzophenanthridine alkaloid (BZDA) biosynthetic pathway, was generated from (*S*)-reticuline by the berberine bridge enzyme (BBE). Jiao et al. (2024) found that targeting *Corydalis yanhusuo* BBE (CyBBE) to the ER using HDEL as the ER routing signal remarkably impacted on (*S*)-SCO production with 206% improvement compared to cytosolic CyBBE. Their work suggests that the ER-targeted CyBBE activity was significantly enhanced due to the more favorable pH of the ER. Higher activity was confirmed in vitro under elevated pH conditions characteristic of the ER lumen (Jiao et al., 2024). Additionally, Jiao et al. (2024) carried out molecular dynamics simulations and discovered that the C-terminal HDEL peptide signal interacted with the BBE enzyme by forming extra hydrogen bonds, thereby improving the compactness and stability of the enzyme. Other instances of ER engineering involved toxicity reduction of hydrogen peroxide by introduction of a mammalian peroxiredoxin IV (mPRDX4) into the ER and expansion of the ER lumen, which improved (*S*)-SCO production to a titer of 113.1 mg/L (Jiao et al., 2024).

As the ER is associated with protein secretory pathways, harnessing the ER as a biochemical factory is a promising approach for the expression of extracellular proteins. Recently, Dong et al. (2024) screened various co-translational translocation signal peptides to express ovalbumin (OVA) in *S. cerevisiae* and found that secretion efficiency improved when OVA was targeted in the ER using the peptide signal from INU1. Later, OVA production was enhanced and ultimately reached 116.3 mg/L by improving protein folding via overexpression of molecular chaperone Kar2, overexpression of disulfide isomerase PDI, and enlarging the ER (Dong et al. 2024). Likewise, de Ruijter et al. (2016) revealed that tailoring the ER through improved protein folding by overexpression of *CPR5*-encoding peptidyl-prolyl isomerase, together with increased ER size boosted the secretion of human antibodies, such as IgG by 10.2-fold compared to wild-type in *S. cerevisiae*.

As biofuel and biochemical production can be leveraged by the ER, engineering approaches that alter ER morphology could further enhance production of native or synthetically ER-localized pathways (Fig. 4). Interestingly, ER expansion facilitated the biosynthesis of terpenoids (Arendt et al., 2017; Emmerstorfer et al., 2015; Kim et al., 2019), human antibody (de Ruijter et al., 2016; Niemelä et al., 2024), ovalbumin (Dong et al., 2024), and BIAs (Jiao et al., 2024), as shown in Table 3. As the MVA-based terpenoid biosynthetic pathways contain several native ER-associated enzymes (e.g., HMG1, HMG2, ERG9, ERG1, and cytochrome P450 reductase (CPR)), modifying ER morphology significantly impacts on terpenoid production by affecting its capacity for enzyme synthesis and post-translational modification (Arendt et al., 2017; Emmerstorfer et al., 2015; Kim et al., 2019). Emmerstorfer et al. (2015) investigated the impact of the ICE2 gene involved in ER biogenesis on the production of trans-nootkatol which was catalyzed by the membrane anchored CYP (cytochrome P450)/CPR system from (+)- valencene in *S. cerevisiae* and *P. pastoris*. Their study demonstrated that overexpression of Ice2 protein led to higher ER membrane proliferation with staggered layers, then improved the stability and activity of CPR enzyme, thus attributing to a more efficient CYP/CPR-based conversion to trans-nootkatol in *S. cerevisiae* (Emmerstorfer et al., 2015). Although CPR expression was also improved by Ice2 upregulation in *P. pastoris*, production of terpenoids was not enhanced. However, its upregulation led to a 2.5-fold increase in conversion of bufuralol to 1-hydroxybufuralol (Emmerstorfer et al., 2015). Arendt et al. (2017) rationally selected the phosphatidic phosphatase, encoded by *PAH1* and catalyzing the formation of triglycerides from phosphatidic acid, as a knock-out (KO) target. They found that deletion of *PAH1* significantly increased ER membrane proliferation and enhanced production of β-amyrin, highly oxygenated sapogenins, and saponin by between 6- and 16-fold Another KO target, OPI1 is a transcriptional factor (TF) that negatively regulates phospholipid biosynthesis and has widely been explored for increasing the ER volume. The OPI1 protein acts as a repressor of the INO2/INO4 TF heterodimer complex. The complex is an activator for transcription of numerous phospholipid synthesis enzymes. Hence, deletion of OPI1 has been previously reported to increase the production of ovalbumin, human antibody, and alkaloid (*S*)-SCO (Dong et al., 2024; de Ruijter et al., 2016; Jiao et al., 2024; Niemelä et al., 2024). Moreover, increasing the ER membrane surface area through knocking out membrane curvature inducing genes like *RTN1*, *RTN2*, and *YOP1* contributed to the improved secretion of IgG (Niemelä et al., 2024). Taken together, quadruple deletion of OPI1, RTN1, RTN2, and YOP1 resulted in 2-fold improvement of ER area, increasing IgG secretion by more than 2-fold (Niemelä et al., 2024). On the other hand, Kim et al. (2019) demonstrated the beneficial effect of ER expansion through upregulation of *INO2*, which encodes a key TF for phospholipid, fatty acid, and sterol synthesis. Overexpression of INO2 alone led to 71-fold higher production of squalene. Introduction of 3 copies of ER-associated tHMG1 further increased squalene titer by 3.6-fold, reaching 634 mg/L (Kim et al., 2019). Specifically, transcriptomic analysis of the INO2-overexpressing strain indicated upregulation of ER-localized chaperones induced by the Ino2 factor, suggesting that proper folding of ER-related proteins was facilitated during ER expansion (Kim et al., 2019). In addition, when OPI1 deletion was applied together with overexpression of the INO2/INO4-TF complex, secretion of ovalbumin protein was enhanced in the final engineered strain (Dong et al., 2024). The improvement is primarily attributed to the dilation of the ER of which expansion might accommodate better protein folding and reduce aggregation (Dong et al., 2024).

## Potentials and Challenges of the Yeast Organelle Engineering

Engineering organelles as microfactories to enhance production of biofuels and biochemicals has been extensively explored in yeasts, namely through mitochondrial, peroxisomal, or endoplasmic reticulum compartmentalization. There is a growing interest in these organelles over the last decade as their native metabolism and their functional capacity align with our growing need for the targeted biosynthetic pathways. This indicates that subcellular compartmentalization of desired biosynthesis pathways within the interested organelles substantially improved the production of the target products. However, maximization of biochemical production via organelle engineering demands an understanding of the interplay between native organelle function and the heterologous biosynthetic pathway. Also, there are remaining challenges of organelle engineering that have to be addressed as described below.

Mitochondria, organelles with high availability of acetyl-CoA, ATP, and cofactors, have been reported to be a subcellular vessel for high level production of several isoprenoids and terpenoids, including isoprene, amorphadiene, limonene, squalene (Kong et al., 2023; Lv et al., 2016; Yao et al., 2018; Yuan & Ching, 2016; Zhu et al., 2021) (Table 1). Nevertheless, mitochondrial production of advanced terpenoids was explored in yeast but yielded suboptimal titers (Dong et al., 2021; Zhang et al., 2020, 2022), indicating there are some bottlenecks to compartmentalizing the entire terpenoid pathways in mitochondria. Low bioproduction is likely caused by the toxicity of phosphorylated MVA pathway intermediates due to their impermeability through the mitochondrial bilayer membrane. The accumulation of phosphorylated metabolites affects cell growth and disturbs the electron transport chain (ETC) in mitochondria, thereby limiting biosynthesis of terpenoids (Zhu et al., 2021). Apart from harnessing dual cytoplasmic and mitochondrial engineering, investigation of mitochondrial transporters might be helpful to tackle the challenge of phosphorylated intermediate impermeability. Considering the toxicity issue, mitochondrial engineering might not be an effective strategy for terpenoids yet remains an enticing production strategy for other platform chemicals such as 3-HP, itaconic acid, and biofuels like fusel alcohols and their ester derivatives. Titers of these molecules could be largely improved using mitochondrial engineering as shown in Table 1.

In contrast to mitochondria, the peroxisome was indicated to be a more favorable organelle for robust production of diverse MVA pathway-based terpenoids and fatty acid-derived chemicals (Table 2) without any negative impacts on cell fitness. Yet, insufficient pools of ATP, NADPH, and, especially, acetyl-CoA stem from poor β-oxidation in yeast. Therefore, providing more energy, cofactor, introducing a peroxisomal acetate-to-acetyl-CoA pathway, and strengthening β-oxidation through accumulating more peroxisomal fatty acids remain promising opportunities to improve production. Another challenge of peroxisome engineering is the small functional capacity of the peroxisome in cells grown on glucose. Engineering of TFs regulating peroxisome biogenesis with overexpression or deletion of rational targets such as peroxins and pexophagy proteins was reported to increase peroxisome number and size (Choi et al., 2022; Grewal et al., 2021; Lin et al., 2023; Zhou et al., 2016), but it has never been confirmed whether functional capacity of peroxisome is genuinely enlarged from these efforts.

Recently, technological development in artificial intelligence and computational algorithms has opened a novel avenue for synthetic biology. Interestingly, an ML-aided pipeline developed by the Dueber group enabled the identification of peroxisome-related genes, whose combinatory overexpression resulted in a 137% increase in peroxisomal functional capacity compared to the wild-type strain, thereby enhancing geraniol production (Baker et al., 2024). Motivated by this study, it is believed that ML would be a powerful tool to assist in the development of organelle engineering approaches to leverage organelle engineering. Even though ER compartmentalization of extended biosynthetic pathway has not been intensively studied, the significance of morphological changes to the ER (e.g., ER proliferation with expanded capacity and staggered sheets) on production of terpenoids, secretory proteins, and lipid-related products suggested that ER engineering can be explored as a depot for other biofuels and biochemicals. Particularly, an enzyme catalyzing the formation of (*S*)-SCO, a precursor of plant-derived products, has been found to have better activity with higher stability in yeast ER owing to local pH and the ER routing peptide, respectively, compared to yeast cytosol (Jiao et al., 2024). This suggests that the ER is a promising subcellular place for the expression of enzymes associated with plant-derived metabolite bioconversion and that ER engineering can be harnessed to enhance natural product generation.

Moreover, simultaneously compartmentalizing the biosynthetic pathway to subcellular organelles could significantly accelerate the product yield as well as reduce the intermediate accumulation. While most studies focused on harnessing the dual cytoplasmic and mitochondrial/peroxisomal engineering (Tables 1 & 2), Ma et al. (2021) employed triple organelles including LD, ER and peroxisome to boost production of lipophilic compound such as astaxanthin. Specifically, simultaneously targeting the fusion enzyme (CrtW-Z) of the astaxanthin pathway to LD, ER, and peroxisome led to a significant improvement in astaxanthin titer at 858 mg/L in flask fed-batch fermentation, compared to that of single/dual organelle engineering (Ma et al., 2021).

**Fig. 5.**
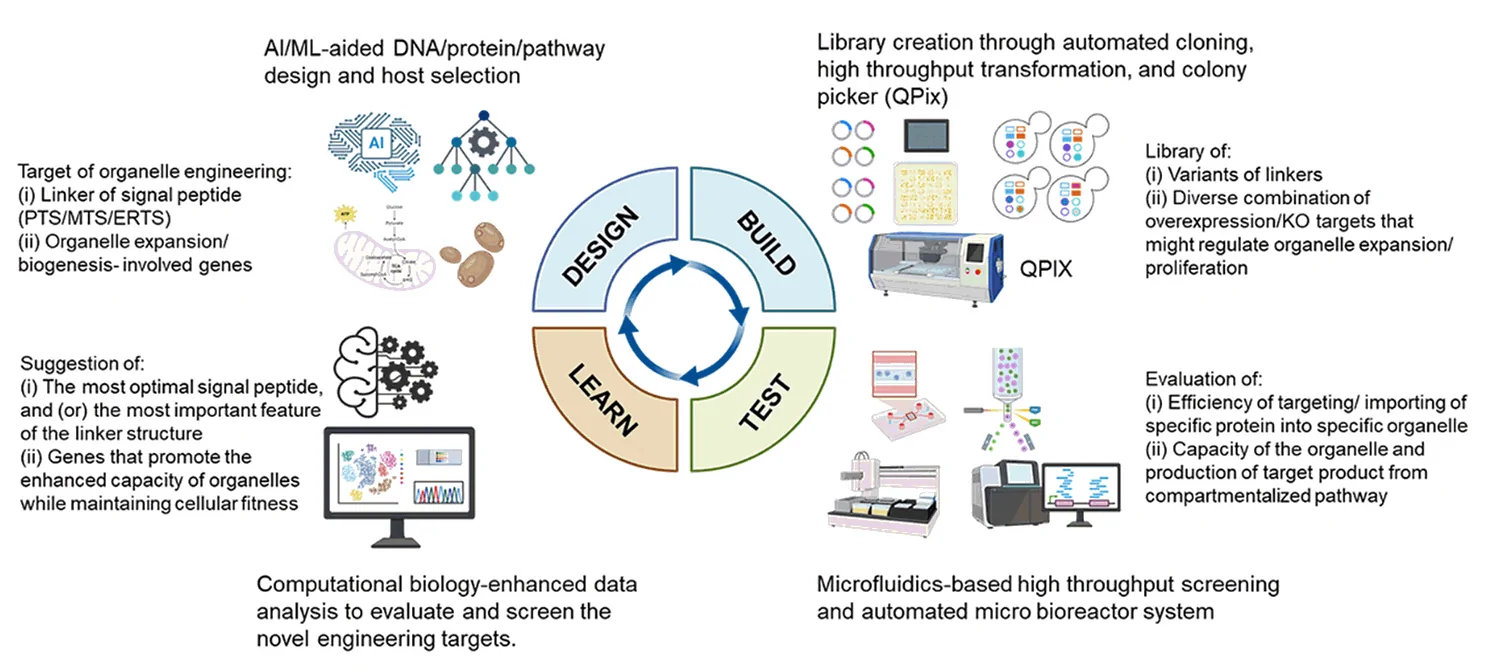
DBTL-based automation pipeline for organelle engineering (created in Biorender). Abbreviations: PTS: peroxisomal targeting sequence, MTS: mitochondrial targeting sequence, ERTS: endoplasmic reticulum targeting sequence, KO: knock-out.

Various high-throughput automation strategies have been developed to accelerate screening and cloning in synthetic biology. Unlocking organelle engineering across numerous yeast species can be significantly expedited by automation pipelines that integrate AI/ML with robotics. In doing so, such pipelines reduce tedious trial-and-error experimentation while integrating into the canonical Design-Build-Test-Learn (DBTL) cycle to enhance productivity of compartmentalized biosynthetic pathways in organelles (Fig. 5). In the case of the organelle signal peptides, a DBTL-based automation pipeline could help to create an enormous library of linker variants. Then, strains could be rapidly engineered through automated cloning, high throughput transformation, selection, and then screening to isolate the best candidate with highest rate of protein importation to a specific organelle (Fig. 5). AI/ML could then be used to help predict signal peptides for other genes and pathways (Radivojević et al., 2020). Moreover, the automation pipeline would serve as a platform for identification of overexpression/KO targets that regulate the morphology/function of the organelles to maximally support the compartmentalized pathways towards the high-level production of biofuels and biochemicals.

## Figures and Tables

**Table 1.** Production of biofuels and biochemicals in yeast species harnessing mitochondrial engineering

Product		Yeast species	MTS type	Single/Dual pathway^*^	Engineering description	Titer	Scale	References
Biochemical	Isoprene	*S. cerevisiae*	COX4	Dual	· Complete MVA pathway in mitochondria (two copies of tHMG1)	2.53 g/L	Bioreactor	Lv et al. (2014^1^, 2016)
					· Diploid strain formation by mating the mitochondrial engineered strain with YXM10 strain^1^			
				Dual	· Isoprene synthase mutant ISPS^LN^ in mitochondria/cytosol	11.09 g/L	Bioreactor	Yao et al. (2018)
					· Complete MVA pathway in mitochondria/cytosol			
					· Additional expression of MVD1 and IDI1 in mitochondria/cytosol			
					· Diploid strain formation by mating 2 mitochondrial/cytosolic strains			
	Amorpha-4,11-diene		COQ3, COX4	Single	· Entire amorphadiene pathway targeted in mitochondria (only ADS tagged with COQ3-MTS)	430 mg/L	Flask	Yuan & Ching (2016)
	8-Hydroxygeraniol		COX4	Single	· Choice of a starting strain JHY65 for its improved respiratory growth and increased mitochondrial stability^2^	227 mg/L;	Bioreactor	Harvey et al. (2018^2^), Yee et al. (2019)
					· The geraniol pathway targeted in mitochondria (using Gg mFPS and ObGES)			
					· Expression of ER-targeted CrG8H			
					· Deletion of OYE2 and OYE3			
	Nepetalactol				· Expression of cytosolic CrGOR and CrISY for nepetalactol synthesis	5.9 mg/L	Tube	
	Linalool		COX4	Dual	· Complete linalool pathway in both mitochondria and cytosol (using ERG20^F96W/N127W^ and CoLIS)	23.45 mg/L	Bioreactor	Zhang et al. (2020)
					· Downregulation of endogenous ERG20			
					· Additional expression of a fusion CoLIS-ERG20^F96W/N127W^ protein in both mitochondria and cytosol.			
				Single	· The entire MVA pathway targeted in mitochondria	23.8 mg/L	Flask	Zhang et al. (2022)
					· Mitochondrial expression of truncated AaLS1 and ERG20^F96W/N127W^			
	Limonene		COX4	Dual	· The entire limonene pathway targeted in mitochondria	2.63 g/L	Bioreactor	Kong et al. (2023)
					· The entire cytosolic limonene pathway with multiple copies of ERG20^F96W/N127W^ and tLimS, and downregulation of ERG20			
					· Enhanced acetyl-CoA pool by knocking out CIT2 and MLS1			
					· Enhancing NADPH pool by overexpressing pentose phosphate (PP) pathway and deleting the NADPH-consuming pathway			
	α-Santalene		MMF1, COX4, HSP60, LSC2, LDP1, ALD4	Single	· Complete α-santalene pathway targeted in mitochondria with additional overexpression of tHMG1	41 mg/L	N/A	Dong et al. (2021)
	Squalene		COX4	Dual	· Complete MVA pathway targeted in mitochondria with additional expression of ERG19	21.1 g/L	Bioreactor	Zhu et al. (2021)
					· Full squalene pathway expressed in cytosol (overexpression only for ERG10, tHMG1, ERG19, IDI1, ERG20, and ERG9)			
					· Expression of ALD6, ASC1, ASC2 and ADH2 for improved cytosolic acetyl-CoA			
					· Down regulation of ERG1			
			COX4	Dual	· Complete MVA pathway targeted in mitochondria with regulation of genes by promoter screening	230 mg/L	Flask	Yanagibashi et al. (2024a)
					· Full squalene pathway in cytosol with additional copies of tHMG1 and ERG12			
			COX4	Single	· Complete MVA pathway targeted in mitochondria	707 nmol/g DCW	Flask	Yanagibashi et al. (2024b)
					· Improved mitochondrial volume by deletion of MDM32			
	β-Carotene		COX4	Single	· Expression of CrtYBXd, CrtIXd, and BTS1 (for β-carotene synthesis)	1609 nmol/g DCW	Flask	
	3-Hydroxypropionate		COX4	Single	· Mitochondrial targeting of mutant ACC1 and dissected MCR	71.09 g/L	Bioreactor	Zhang et al. (2023)
					· Overexpression of IDP1 and POS5 for improved NADPH pool in mitochondria			
	Itaconic acid	*A. niger*	ICDA, ACOA	Dual	· Itaconic acid pathway targeted in mitochondria (AtCadA and AnAcoA)	1.4 g/L	Flask	Blumhoff et al. (2013)
					· Expression of itaconic acid pathway in cytosol (AtCadA and EcAcnA)			
	Acetoin	*C. glabrata*	COX4	Single	· Acetoin pathway targeted in mitochondria (BsALS and BaALDC)	3.26 g/L	Flask	Li et al. (2015)
					· Overexpression of mitochondrial pyruvate carrier (ScMPC)			
Biofuel	Isobutanol	*S. cerevisiae*	COX4	Single	· Mitochondrial targeting of ARO10, LlKiVD and AdhA^RE1^, ADH7	635 mg/L	Tube	Avalos et al. (2013)
					· Overexpression of mitochondrially native genes ILV2, ILV5, ILV3			
				Single	· Mitochondrial targeting of ARO10, LlAdhA^RE1^	1.245 g/L	Plate	Hammer & Avalos (2017b)
					· Deletion of BAT1 and ILV6			
				Single	· Mitochondrial targeting of ARO10, LlAdhA^RE1^	8.49 g/L	Bioreactor	Zhao et al. (2018)
					· Deletion of BAT1			
	2-Methyl-1-butanol			Single	· Optogenetic regulation of ILV2 and PDC1	2.38 g/L	Bioreactor	
	Isopentanol		COX4, CD9, COX6	Single	· Mitochondrial targeting of LEU4 mutant, LEU1, LEU2	1.24 g/L	Plate	Hammer et al. (2020)
					· Deletion of LEU4, LEU9, BAT1 and OAC1			
	n-Butanol		COX4, CYB2, CAT2	Single	· Mitochondrial targeting of LEU4, LEU1, LEU2 and citramalate synthase (LiCim)	1.05 g/L	Bioreactor	Shi et al. (2016)
					· Overexpression of LEU9, cysteine desulfurase NFS1, ARO10 and ADH7			
	Fatty acid alkyl ester		COX4	Single	· Mitochondrial targeting of ARO10 and ADH7 for improved isobutanol production	230 mg/L	Flask	Teo et al. (2015)
					· Overexpression of two wax ester synthase Ms Ws2 and Maqu_0168			
					· Deletion of OPI1 and RPD3			
	Isobutyrate		COX4	Dual	· Mitochondrial targeting of ARO10 and ADH7, and ATF1	260.2 mg/L	Tube	Yuan et al. (2016)
	3-Methyl-1-butyl acetate				· ATF1 overexpression in cytosol	296.1 mg/L		
	2-Methyl-1-butyl acetate				· Overexpression of PYC2, MDH2, MAE1	289.6 mg/L		

* The single pathway refers to the entire biosynthetic pathway or partial downstream pathway, compartmentalized either in the cytosol or in a subcellular organelle; whereas the dual pathway refers to the entire biosynthetic pathway that is expressed in both the cytosol and a subcellular organelle. Gg: *Gallus gallus*; Ob: *Ocimum basilicum*; Cr: *Catharanthus roseus*; Co: *Cinnamomum osmophloeum*; Aa: *Actinidia arguta*; Ll: *Lactococcus lactis*; Li: *Leptospira interrogans*; Ms: *Marinobacter* sp.; Bs: *Bacillus subtilis*; Ba: *Bacillus amyloliquefaciens*; Sc: *Saccharomyces cerevisiae*; At: *Aspergillus terreus*; An: *Aspergillus niger*; Ec: *Escherichia coli*

**Table 2.** Production of biofuels and biochemicals in yeast species harnessing peroxisome engineering

Product		Yeast species	PTS type	Single/Dual pathway^*^	Engineering description	Titer	Scale	References
Biochemical	Geraniol	*S. cerevisiae*	SKL	Single	· Compartmentalization of AgGPPS2 and ObGES in peroxisome	2.75 mg/L	Plate	Gerke et al. (2020)
					· Deletion of PEX30 and PEX32 for increased peroxisome number			
					· Expression of truncated α-arrestin-like adaptor Bul1 for high tolerance to geraniol			
					· The entire geraniol pathway targeted in peroxisome (using ERG20^N127W^and ObGerS) with episomal plasmids	5.52 g/L	Bioreactor	Dusséaux et al. (2020)
				Dual	· Machine learning (ML)-aided identification of the MVA pathway’s critical gene	120 mg/L	Flask	Mukherjee et al. (2022)
					· The MVA pathway targeted in peroxisome			
					· The dual MVA platform strain (diploid) created by mating peroxisomal and cytosolic strains			
					· Overexpression of a fusion tObGES-ERG20^WW^			
			ePTS1	Single	· The entire geraniol pathway targeted in peroxisome (using ERG20^F96W, N127W^ and tVoGES)	9.5 g/L	Flask	Baker et al. (2024)
					· ML-guided screen of peroxisome complexes for improved peroxisome capacity			
		*P. pastoris*	ePTS1, PTS of TAL1-2	Single	· HTS-based screen of 25 putative PTS, followed by directed evolution of selected 3 PTS1 with high targeting efficiency	20 mg/L	Plate	Ye et al. (2024)
					· Peroxisomal compartmentalization of geraniol pathway (only tCrGES tagged with newly identified PTS; the remaining proteins tagged with ePTS1).			
	R-Limonene	*R. toruloides*	SKL, PTS2 (C4N11 of 3-KT)	Dual	· Peroxisomal targeting of the entire limonene pathway (two copies of NPPS::LS)	1.05 g/L	Flask	Gao et al. (2024a)
					· Peroxisomal targeting of Nph17 and overexpressing Nph17 and ACCT for improved acetoacetyl-CoA in peroxisome and cytosol, respectively			
					· Cytosolic overexpression of NPPS::LS			
					· Overexpression of ACL for improved cytosolic acetyl-CoA			
		*S. cerevisiae*	SKL	Single	· Peroxisomal targeting of the entire biosynthetic pathway targeted with episomal plasmids.	2.58 g/L	Bioreactor	Dusséaux et al. (2020)
					· R-limonene: ClLimS			
	α-Pinene				· α-Pinene: PtPinS	69.22 mg/L	Flask	
	Sabinene				· Sabinene: SpSabS	32.32 mg/L		
	Camphene				· Camphene: _1_SeCamS	5.77 mg/L		
	8-Hydroxygeraniol				· 8-Hydroxygeraniol: ObGerS, CrG8OH, CrCPR	25.11 mg/L		
	Canabigerolic acid (CBGA)				· CBGA: CsPT4	0.82 mg./L		
	Linalool		ePTS1	Dual	· Improving catalytic efficiency toward GPP of linalool synthase through site-directed mutagenesis	2.6 g/L	Bioreactor	Zhou et al. (2023)
					· Complete linalool pathway targeting to peroxisome			
					· Overexpression of tHMG1, IDI1, ERG20^F96W, N127W^, and McLIS with repression of native ERG20 for cytosolic linalool pathway			
					· Mating peroxisomal and cytosolic strain to form the dual strain (diploid)			
	β-Myrcene		SKL	Dual	· Source screening of β-myrcene synthase, with truncated QiMS as the best candidate	142.64 mg/L	Bioreactor	Shu et al. (2024)
					· Peroxisome localization of fusion enzyme tQiMS^D436N^-ERG20^WW^			
					· Overexpression of tHMG1 and IDI in the cytosolic MVA pathway and replacement of native ERG20 by ERG20^W^			
					· Enhancing cytosolic acetyl-CoA by overexpressing ADH2 and ALD6			
	α-Humulene	*S. cerevisiae*	ePTS1	Dual	· Compartmentalization of entire α-humulene pathway in peroxisome	1.73 g/L	Bioreactor	Zhang et al. (2020)
					· Overexpression of tHMG1 and ERG20 in cytosol			
					· Down regulation of ERG9 by replacing its native promoter by HXT1p and adding PEST sequence			
					· Additional overexpression of cytosolic ZzZSS1 in rDNA sites			
			SKL	Dual	· ML-guided dual MVA platform strain (diploid) created by mating peroxisomal and cytosolic strains	~22.5 mg/L	Flask	Mukherjee et al. (2022)
					· Overexpression of cytosolic ERG20 and ZzZSS1			
			oPTS1*	Dual	· Develop an orthogonal transport system ScPEX5*-oPTS1*	17.33 g/L	Bioreactor	Zhang et al. (2024)
					· rDNA integration of the entire cytosolic and peroxisomal α-humulene pathway (ZSS1 as humulene synthase)			
					· Overexpression of ANT1, IDP2, IDP3 (rDNA integration) for improved ATP and NADPH pool in peroxisome			
					· Down regulation of ERG9 by replacing its native promoter by HXT1p and adding PEST sequence			
		*Y. lipolytica*	PTS (GGGSSKL)	Single	· Localization of entire α-humulene pathway in peroxisome (NADH-HMG1 as HMG-CoA reductase; AcHS2 as humulene synthase)	3.2 g/L	Bioreactor	Guo et al. (2021)
					· Increasing ATP supply in peroxisome by overexpression of ANT1			
					· Adjusting the copy numbers of rate-limiting enzymes (one more copy of peroxisomal targeting NADH-HMG1 and AcHS2)			
					· Upregulation of β-oxidation by overexpressing POT1			
					· Oleic acid as a possible C source for α-humulene accumulation			
			PTS (GGGSSKL)	Dual	· Iterative integration of the entire cytosolic and peroxisomal α-humulene pathway (AcHS2 as humulene synthase)	21.7 g/L	Bioreactor	Guo et al. (2022)
					· Mediating copy number of cytosolic tHMG1 and AcHS2			
					· Improving cytosolic acetyl-CoA by overexpression of CoA‐acetylating aldehyde dehydrogenase (EcAAD)			
					· Repression of ERG9 expression using Cu^2+^ repressible promoter			
		*Candida tropicalis*	SKL	Single	· Multiple overexpression of the entire peroxisomal α-humulene pathway (double copies of the pathway)	2.42 mg/L	Flask	Zhang et al. (2022)
	α-Bisabolene	*P. pastoris*	SKL and LARF	Single	· Peroxisome isolation of the entire α-bisabolene pathway with fusion enzyme of FPPS-AgBIS; upregulation of acetyl-CoA-to-mevalonate pathway; and additional copy of separated FPPS and AgBIS	1.1 g/L	Flask	Gao et al. (2024b)
	α-Farnesene		ePTS1	Dual	· Overexpression of tHMG1, IDI1, ERG20 and MdAFS for cytosolic α-farnesene pathway	2.56 g/L	Flask	Liu et al. (2021)
					· Improving cytosolic acetyl-CoA pool by introducing pyruvate dehydrogenase (Ec cytoPDH) and ATP-dependent citrate lyase (YlACL)			
					· Introduction of IUP pathway and downstream genes (2 copies of IDI, ERG20, MdAFS) into peroxisome			
	β-Amyrin	*S. cerevisiae*	ePTS1	Dual	· Peroxisome compartmentalization of squalene pathway	2.6 g/L	Bioreactor	Du et al. (2022)
					· Double copies of downstream β-amyrin pathway (ERG1 and GgbAS1) in cytosol			
					· Deregulation of ERG7			
	Squalene		ePTS1	Dual	· The entire squalene pathway targeted in peroxisome with an additional copy of tHMG1 and IDI1	11 g/L	Bioreactor	Liu et al. (2020)
					· Deletion of GPD1 and GPD2			
					· Overexpression of ANT1, IDP2 and IDP3 for improved ATP and NADPH pool in peroxisome			
					· ACS1, YlACL1 and YlACL2 targeted to peroxisome for improved peroxisomal acetyl-CoA			
					· Diploid strain formation by mating two peroxisomal and cytosolic strains			
			SKL	Dual	· The dual MVA platform strain (diploid) created by mating peroxisomal and cytosolic MVA strains	300 mg/L	Flask	Mukherjee et al. (2022)
					· Overexpression of ERG9 and ERG20			
					· Prevention of squalene degradation by supplementation of terbinafine			
		*Y. lipolytica*	SKL	Dual	· The complete squalene pathway targeted in peroxisome	32.8 g/L (on glucose)/ 31.6 g/L (on acetate)	Bioreactor	Ma et al. (2024)
					· Enhanced TAG-derived free fatty acids in peroxisome through overexpressing TlTGL, DGA1 and ACC1			
					· Improved cytosolic acetyl-CoA pool by overexpressing PYC1, YHM2 and MmACL			
					· Upregulated β-oxidation by overexpressing POX2, MFE1 and POT1			
					· Increased size/number of peroxisomes by overexpressing PEX10			
					· Introduction of Acetyl-CoA shortcut in the peroxisome by targeting _2_SeACS1^L641P^.			
	Lycopene	*P. pastoris*	SKL	Single	· Peroxisomal compartmentalization of lycopene biosynthetic pathway (CrtE, CrtB, CrtI)	73.9 mg/L	Bioreactor	Bhataya et al. (2009)
	Astaxanthin	*Y. lipolytica*	SKL	Single	· Peroxisomal targeting of the fusion enzyme PsCtrW-HpCrtZ	58.7 mg/L	Flask	Ma et al. (2021)
	Protopanaxadiol	*S. cerevisiae*	PTS1, PTS2	Single	· Regulating number and size of peroxisome through deletion of PEX11 and ATG36, and overexpression of PEX34	4.1 mg/L	Flask	Choi et al. (2022)
					· Peroxisomal compartmentalization of protopanaxadiol pathway (including tHMG1, ERG9, PgErg1, PgDs, PgPpds, and PgCpr)			
	(S)-Norcoclaurine		ePTS1	Single	· Targeting norcoclaurine synthase (NCS) in peroxisome	~7 mg/L	Plate	Grewal et al. (2021)
					· Controlling peroxisome biogenesis by engineering transcriptional factors ADR1, OAF1 and PIP2			
	Ophiobolins U		PTS of PEX15	N/A	· Peroxisomal surface display of CAT2 for improved cytosolic acetyl-CoA	128.9 mg/L	Flask	Zhang et al. (2024)
	Indole-3-acetic acid	*K. marxianus*	PTS of PEX15	Single	· Peroxisomal surface display of tryptophan-2-monooxygenase *Ps*IaaM and indole-acetamide hydrolase PsIaaH	~61.25 mg/L	Tube	Bassett et al. (2024)
					· Controlling peroxisome number and size by overexpression of PEX19			
	Triacetic acid lactone (TAL)		PTS of PEX15	Single	· Peroxisomal surface display of CAT2, ACC1 and Gh2-PS	~ 1.1 g/L	Tube	
					· Controlling peroxisome number and size by overexpression of PEX11			
		*S. cerevisiae*	PTS of PEX15	Single	· Peroxisomal surface display of CAT2, ACC1^S1157A^ and Gh2-PS	0.77 g/L	Tube	Yocum et al. (2022)
			pPTS (PTS of Psc60)	Dual	· Cytosolic expression and peroxisomal targeting of Gh2-PS and ACC1^S686A, S659A, S1157A^	0.14 g/L	Plate	Lin et al. (2023)
					· Increasing peroxisome proliferation by overexpression of ADR1, OAF1 and PIP2			
	Flaviolin		ePTS1	Single	· Peroxisomal targeting of Sac_RppA and ACC1^S686A, S659A, S1157A^	N/A	N/A	
	Desmethylxanthohumol (DMX)		N/A	Single	· Compartmentalization of the complete MVA pathway in peroxisome	~62.5 µg/L	Flask	Yang et al. (2024)
					· Peroxisomal targeting of ACC1 and naringenin chalcone and DMX biosynthetic genes			
	Amino acid (Lysine, Histidine)	*S. japonicus*	PTS1 and PTS2	N/A	· Peroxisomal targeting of GPD2, LYS3 and HIS2 enzymes	N/A	N/A	Gu et al. (2023)
					· Regulating peroxisome size with PEX5 mutant and PEX11			
	Penicillin	*Hansenula polymorpha*	SKL	Single	· Peroxisomal targeting of penicillin pathway’s bottom genes, including PcIAT and PcPCL	1 mg/L	Plate	Gidijala et al. (2009)
	PHA	*S. cerevisiae*	PTS of *Bn*ICL	Single	· Peroxisomal targeting of PaPHAC1 synthase	0.45 g/g DCW	Flask	Poirier et al. (2001)
Biofuel	Fatty acid ethyl esters (FAEEs)	*Y. lipolytica*	SKL	Single	· Peroxisomal targeting of wax ester synthase (AbAtfA)	110.9 mg/L	Flask	Xu et al. (2016)
	Fatty alcohols	*S. cerevisiae*	PTS2	Single	· Peroxisomal targeting of fatty acyl-CoA reductase (TaFAR)	1.3 g/L	Tube	Sheng et al. (2016)
					· Increasing peroxisomal importing rate by overexpression of PEX7			
					· Improved malonyl-CoA pool by overexpression of ACC1			
			PTS2	Single	· Peroxisomal targeting of fatty acyl-CoA reductase *Ma*FAR in fatty acyl-CoA overproducing strain	193 mg/L	Flask	Zhou et al. (2016)
	Alkanes		PTS1 and PTS2	Single	· Peroxisomal targeting of free fatty acid (FFA) pathway including MmCAR, AnNPGA, _3_SeADO, ferredoxin _3_SeFd and ferredoxin reductase _3_SeFNR	~ 3.5 mg/L	Flask	
					· Increasing fatty acid production by deleting HFD1 and POX1			
					· Increasing peroxisome population by deletion of PEX31, 32 and overexpression of PEX34			
	Olefins		PTS2		· Peroxisomal targeting of OleT with flavodoxin EcFldA and flavodoxin reductase EcFNR	~ 0.20 mg/L	Flask	
	α-Alkenes	*P. pastoris*	PTS1 and PTS2	Single	· Peroxisomal targeting of decarboxylase PfUndB together with cofactor protein putidaredoxin–putidaredoxin reductase PpPdr/Pdx	1.6 mg/L	Flask	Cai et al. (2022)

* The single pathway refers to the entire biosynthetic pathway or partial downstream pathway, compartmentalized either in the cytosol or in a subcellular organelle; whereas the dual pathway refers to the entire biosynthetic pathway that is expressed in both the cytosol and a subcellular organelle. Yl: *Yarrowia lipolytica*; Ob: *Ocimum basilicum*; Ag: *Abies grandis*; Vo: *Valeriana officinalis*; Tl: *Thermomyces lanuginosus*; Mm: *Mus musculus*; HTS: *high throughput screening*; Cr: *Catharanthus roseus*; Cl: *Citrus limon*; Pt: *Pinus taeda*; Sp: *Salvia pomifera*; _1_Se: *Solanum elaeagnifolium*; Cs: *Cannabis sativa*; _2_Se: *Salmonella enterica*; Ps: *Paracoccus* sp.; Hp: *Haematococcus pluvialis*; Zz: *Zingiber zerumbet*; Sc: *Saccharomyces cerevisiae*; At: *Arabidopsis thaliana*; Ac: *Aquilaria crassna*; Ec: *E. coli*; Gg: *Glycyrrhiza glabra*; Qi: *Quercus ilex*; Md: *Malus domestica*; Pg: *Panax ginseng*; Sac: *Streptomyces aculeolatus* ; Gh: *Gerbera hybrid*; Ab: *Acinetobacter baylyi ADP1*; Ta: *Tyto alba*; Ma: *Marinobacter aquaeolei*; _3_Se: *Synechococcus elongates*; Mm: *Mycobacterium marinum*; An: *Aspergillus nidulans* ; Pf: *Pseudomonas fluorescens*; Pp: *Pseudomonas putida*; Pc: *Penicillium chrysogenum*; Ps: *Pseudomonas savastanoi*; Pa: *Pseudomonas aeruginosa*; Bn: *Brassica napus*; NPPS::LS: the chimera of NPP synthase from *Solanum lycopersicum* and limonene synthase from *Citrus limon*

**Table 3.** Endoplasmic reticulum engineering of yeasts for biofuels and biochemicals production

Product		Yeast species	ER engineering	Titer	Scale	References
Biochemical	Opiate (morphine)	*S. cerevisiae*	· ER targeting of NADPH-dependent aldo-keto reductase PsCOR1.3 with ER signal peptide from CNE1	3.1 mg/L	Plate	Thodey et al. (2014)
	Ceramide NS	*S. cerevisiae*	· ER targeting of human sphingolipid desaturase (hDES1) with ER retention signal KKEK from SUR2	N/A	N/A	Murakami et al. (2015)
	Trans-nootkatol	*S. cerevisiae*	· Regulating ER membrane proliferation by overexpression of ICE2	30 mg/L	Flask	Emmerstorfer et al. (2015)
	1-Hydroxybufuralol	*P. pastoris*		N/A	N/A	
	β-Amyrin	*S. cerevisiae*	· Expansion of ER by deleting the phosphatidic acid phosphatase PAH1	N/A	N/A	Arendt et al. (2017)
	Medicagenic acid			27.1 mg/L	Flask	
	Oleanane-type sapogenin			N/A	N/A	
	Squalene	*S. cerevisiae*	· Expansion of ER by overexpression of transcriptional factor INO2	634 mg/L	Flask	Kim et al. (2019)
	Protopanaxadiol	*S. cerevisiae*	· Controlling gene dosage of tHMG1 (3 copies) for squalene production	12.1 mg/L	Flask	
	Astaxanthin	*Y. lipolytica*	· ER targeting of the fusion enzyme β-carotene ketolase-hydroxylase (PsCtrW-HpCrtZ) with ER signal peptide KDEL	53.2 mg/L	Flask	Ma et al. (2021)
	Human antibody (IgG)	*S. cerevisiae*	· Expansion of ER by deleting lipid-regulator OPI1	126 µg/L	Plate	de Ruijter et al. (2016)
			· Overexpression of peptidyl-prolyl isomerase CPR5 for enhanced secretion			
			· Modification of ER morphology and size by deleting lipid-regulator gene *OPI1* and ER membrane curvature genes *RTN1*, *RTN2* and *YOP1*	79 ng/mL	Plate	Niemelä et al. (2024)
	Ovalbumin	*S. cerevisiae*	· Expression of OVA using signal peptide from INU1	116.3 mg/L	Bioreactor	Dong et al. (2024)
			· Co-expression of chaperone Kar2 and disulfide isomerase PDI for improved folding environment in ER			
			· Expansion of ER by deleting OPI1 and overexpressing transcriptional factors INO2 and INO4			
	*S-*Scoulerine	*S. cerevisiae*	· ER targeting of berberine bridge enzyme (CyBBE) with ER C-terminal HDEL signal peptide (CyBBE_ERTS)	113.1 mg/L	Flask	Jiao et al. (2024)
			· ER targeting of mammalian peroxiredoxin IV (PRDX4) with N- and C-terminal ERTS (α- mPRDX4_ERTS) for reducing toxicity of hydrogen peroxide			
			· Expansion of ER by deleting transcriptional regulator OPI1 of phospholipid biosynthesis			
Biofuel	Fatty acid ethyl esters (FAEEs)	*Y. lipolytica*	· ER targeting of wax-ester synthase (AbAtfA) with ER peptide signal KDEL	136.5 mg/L	Flask	Xu et al. (2016)
	Fatty alkane		· ER targeting of fatty acyl-CoA reductase (AbACR1)-aldehyde deformylating oxygenase (PmADO) gene cluster with ER signal peptide KDEL	16.8 mg/L	Flask	
	Fatty alcohol		· ER targeting of fatty acyl-CoA reductase (AbACR1) and aldehyde reductase (EcAHR) with ER signal peptide KDEL	49.2 mg/L	Flask	

Ps: *Papaver somniferum*; Cy: *Corydalis yanhusuo*; Ab: *Acinetobacter baylyi*; Ps: *Paracoccus* sp.; Hp: *Haematococcus pluvialis*; Pm: *Prochlorococcus marinus*.
